# Screening for inborn errors of metabolism in high-risk children: a 3-year pilot study in Zhejiang Province, China

**DOI:** 10.1186/1471-2431-12-18

**Published:** 2012-02-24

**Authors:** Xinwen Huang, Lili Yang, Fan Tong, Rulai Yang, Zhengyan Zhao

**Affiliations:** 1 Department of Genetics and Metabolism, Children's Hospital, Zhejiang University School of Medicine, Hangzhou, China; 2 Laboratory Center, Children's Hospital, Zhejiang University School of Medicine, Hangzhou, China; 3 57 zhuganxiang, Hangzhou 310003, China

**Keywords:** Tandem mass spectrometry, Inborn errors of metabolism, Aminoacidemia, Fatty acid oxidation disorders, Organic acidemia

## Abstract

**Background:**

Tandem mass spectrometry (MS/MS) has been available in China for 8 years. This technique makes it possible to screen for a wide range of previously unscreened inborn errors of metabolism (IEM) using a single test. This 3-year pilot study investigated the screening, diagnosis, treatment and outcomes of IEM in symptomatic infants and children.

**Methods:**

All children encountered in the Newborn Screening Center of Zhejiang Province during a 3-year period with symptoms suspicious for IEM were screened for metabolic diseases. Dried blood spots were collected and analyzed by tandem mass spectrometry. The diagnoses were further confirmed by clinical symptoms and biochemical analysis. Neonatal intrahepatic cholestasis caused by citrin deficiency, ornithine transcarbamylase deficiency and primary carnitine deficiency were confirmed by DNA analysis.

**Results:**

A total of 11,060 symptomatic patients (6,720 boys, 4,340 girls) with a median age of 28.8 months (range: 0.04-168.2 months) were screened. Among these, 62 were diagnosed with IEM, with a detection rate of 0.56%. Thirty-five were males and 27 females and the median age was 3.55 months (range 0.07-143.9 months). Of the 62 patients, 27 (43.5%) had aminoacidemias, 26 (41.9%) had organic acidemias and nine (14.5%) had fatty acid oxidation disorders.

**Conclusions:**

Because most symptomatic patients are diagnosed at an older age, mental retardation and motor delay are difficult to reverse. Additionally, poor medication compliance reduces the efficacy of treatment. More extensive newborn screening is thus imperative for ensuring early diagnosis and enhancing the treatment efficacy of IEM.

## Background

The use of tandem mass spectrometry (MS/MS) in newborn screening makes it possible to screen for a wide range of previously unscreened inborn errors of metabolism (IEM) using a single test [1] The disease profile includes aminoacidemias, fatty acid oxidation (FAO) disorders and organic acidemias. Early screening and diagnosis may help to decrease mortality and morbidity rates in children with IEM.

MS/MS has been available in China for 8 years, since its first use for IEM detection in Shanghai in 2003. However, only five cities or provinces (Beijing, Shanghai, Wuhan, Guangdong Province and Zhejiang Province) in China currently screen for IEM using MS/MS in symptomatic infants or newborns. The Newborn Screening Center of Zhejiang Province is the largest screening center in China, and initially implemented MS/MS for screening 26 treatable metabolic disorders in symptomatic infants in 2008, and expanded this to newborn screening in 2009. However, MS/MS newborn screening is not currently mandatory, and only 10% of annual births in Zhejiang Province are screened [2] Samples from symptomatic patients with suspected IEM from throughout the province and neighboring provinces are sent to the Newborn Screening Center of Zhejiang Province. The cost of MS/MS screening in symptomatic infants is 390 RMB (around $59.72). None of the fees for screening, diagnosis or treatment are covered by medical insurance.

In this 3-year pilot study, we investigated the screening, diagnosis, treatment and outcomes of IEM in symptomatic infants and children.

## Methods

### Study subjects

All symptomatic children at the Newborn Screening Center of Zhejiang Province during a 3-year period were screened for metabolic diseases. Symptomatic children included those with symptoms suspicious for IEM including metabolic acidosis, jaundice, hepatosplenomegaly, recurrent vomiting, hypoglycemia, hyperammonemia, mental retardation of unknown cause, language retardation, seizures and unconsciousness. Patients with perinatal brain injury, central nervous system infections, brain trauma, toxicology, tumors and chromosome anomalies were excluded from the study. This study was approved by the Ethical Committee of Children's Hospital, Zhejiang University School of Medicine. Parent consents were obtained for publication of the children's clinical details.

### Mass spectrometry materials and equipment

Dried blood spots were collected from all patients on Whatman 903 filter paper (Wallac OY Turku, Finland). Blood spots were analyzed using electrospray ionization liquid chromatography-mass spectrometry (LC-MS) with a Quattro Micro API (Waters, MA, USA) tandem mass spectrometer. All procedures for sample preparation and MS analysis were performed NeoGram AAAC Spectrometry kit (Perkin Elmer, MA, USA) according to the manufacturer's protocol. Briefly, single disks were punched from each dried blood spot using an automatic or manual 3-mm punch. One disk was added per well. It was recommended to use the first 2-14 wells as blanks for every plate, to allow the LC system and mass spectrometer to synchronize. Using a multichannel pipette and reverse pipetting, 90 μl of the daily working extraction solution (containing a mixture of the respective stable-isotope-labeled internal standards) was added to each well. The plate was shaken and incubated, and 60 μl of the solution was then transferred to a V-bottomed, heat-resistant microplate and evaporated to dryness on a heating block at 55°C under nitrogen. A volume of 50 μl of 3.0 N butanolic HCl was pipetted into each sample and incubated for 30 minutes at 60°C. After incubation, the solution was again evaporated to dryness on a heating block at 55°C under nitrogen. Derivatized samples were then reconstituted with 75 μl of NeoGram AAAC reconstitution solution and the plate was covered with aluminum foil, followed by incubation at 27°C for 10 minutes. The plate was finally placed in the autosampler for testing.

Eight amino acids, including citrulline (Cit), phenylalanine (Phe), methionine (Met), tyrosine (Tyr), valine (Val), leucine (Leu), arginine (Arg), ornithine (Orn), and 20 acylcarnitines were analyzed. The acylcarnitines analyzed included C0, C2, C3, C3DC, C4, C5, C5:1, C5DC, C5OH, C6, C8, C10, C12, C14, C16, C18, C18:1, C16OH, C18OH, and C18:1OH. The indexes and related disorders are shown in Table 1.

**Table 1:** MS/MS screening profiles

MS/MS analytes	Cut-off value (μmol/l)	Possible disorder(s)
Amino acids		
↑PHE	> 103.18	Phenylketonuria
↑PHE/TYR	> 1.43	BH4 deficiency
↑MET	> 64.11	Homocystinuria
↑MET/PHE	> 0.92	
↑LEU	> 327.51	Maple syrup urine disease
↑LEU/PHE	> 4.85	
↑VAL	> 433.56	
↑TYR	> 305.87	Tyrosinemia
↑TYR/PHE	> 4.00	
↑CIT	> 37.35	Citrullinemia
↑CIT/PHE	> 0.70	Neonatal intrahepatic cholestasis caused by citrin deficiency
↑ARG	> 40.77	Argininemia
↑ARG/ORN	> 0.70	
↓CIT	< 6.05	Ornithine transcarbamylase deficiency
↑ORN	> 393.08	
Organic Acids		
↑C3	> 4.33	Methylmalonic acidemia
↑C3/C2	> 0.20	Propionic acidemia
± C4DC	> 1.92	
↑C3DC	> 0.14	Malonic acidemia
↑C3DC/C4	> 0.80	
↑C4	> 0.92	Glutaric acidemia type II (multiple acyl-CoA dehydrogenase
↑C5	> 0.69	deficiency)
↑C8	> 0.33	
↑C14	> 0.59	
↑C16	> 6.13	
↑C12	> 0.47	
↑C5	> 0.69	Isovaleric acidemia
↑C5/C2	> 0.03	
↑C5DC	> 0.14	Glutaric acidemia type I
↑C5DC/C8	> 2.50	
↑C5OH	> 0.73	3-methylcrotonyl-CoA carboxylase deficiency
↑C 5OH/C3	> 0.13	3-OH-3-methylglutaryl-CoA lyase deficiency
(± C5:1)	> 0.12	Multiple carboxylase deficiency
(± C6DC)	> 0.14	
(± C3)	> 4.33	
↑C5:1(± C5OH)	> 0.12	β-Ketothiolase deficiency
Fatty acid oxidation defects		
↓ C0	< 15.0	Primary carnitine deficiency
↓C2	< 9.82	
↑C0	> 90.0	Carnitine palmitoyltransferase I deficiency
↑C0/(C16 +	> 30.00	
C18)		
↓C16	< 0.75	
↑C4	> 0.92	Short-chain acyl-CoA dehydrogenase deficiency
↑C4/C2	> 0.40	
↑C8	> 0.33	Medium-chain acyl-CoA dehydrogenase deficiency
↑C8/C10	> 0.37	
(± C6	(> 0.33	
C10:1)	> 0.29)	
↑C14:1	> 0.39	Very long-chain acyl-CoA dehydrogenase deficiency
↑C14:1/C16	> 0.29	
(± C14		
C16, C18:1)		
↑C16	> 6.13	Carnitine palmitoyltransferase II deficiency
↑C18	> 2.68	Carnitine-acylcarnitine translocase deficiency
↑C18:1	> 2.7	
↑C16OH	> 0.21	Long-chain hydroxyacyl-CoA dehydrogenase deficiency
↑C18OH	> 0.17	Trifunctional protein deficiency
↑C18:1OH	> 0.15	

### Cut-off values

The borderline cut-off values were determined by a pilot study of acylcarnitines and amino acids in 12,720 full-term newborns. The cut-off value was set at four standard deviations (SDs) above or lower the mean value (Table 1). All cut-offs were modified in light of the results of further analyses and more clinical data. Patients were referred immediately for confirmatory tests if the results were above the cut-off value. Repeat analysis of the same sample was performed when the results were outside the cut-off value. Patients were referred for confirmatory tests if the second analysis was also outside the cut-off value.

### Confirmatory tests

Confirmatory tests included repeat MS/MS analysis, urinary organic acid analysis by GC-MS, amino acid analysis, routine blood analysis, biochemistry, blood gas analysis, blood glucose and ammonia tests, blood homocysteine, lactate and pyruvate tests, urine acetone tests, biotin, biotin enzyme profile and DNA analysis. Uropterin profile analysis and aminoacidemias were confirmed by blood amino acid profile analysis and urine GC/MS analysis; neonatal intrahepatic cholestasis caused by citrin deficiency (NICCD), ornithine transcarbamylase (OTC) deficiency and primary carnitine deficiency (PCD) were confirmed by DNA analysis; blood dihydropteridine reductase activity test and BH_4 _loading test were performed to subclassify hyperphenylalaninemia (HPA) into phenylketonuria (PKU) and BH_4 _deficiencies. The diagnosis of organic acidemias depended mainly on urine GC/MS analysis, and multiple carboxylase deficiency (MCD) was confirmed by biotin and biotin enzyme profile analysis. PCD was confirmed by genetic analysis. All the tests were done in our laboratory, except for the genetic analyses that were performed in the Genetic Metabolic Laboratory of the Women and Children's Hospital of Beijing University, Shanghai Genetic and Metabolic Institute, and the Genetic Metabolic Laboratory of Tongji Medical School, Huazhong University of Science and Technology.

## Results

A total of 11,060 symptomatic patients (6,720 boys, 4,340 girls) with a median age of 28.8 months (range: 0.04-168.2 months) were screened. Among the screened patients, 62 were diagnosed with IEM, with a detection rate of 0.56%. Thirty-five were males and 27 females, and the median age was 3.55 months (range: 0.07-143.9 months). Of the patients, 27 (43.5%) had aminoacidemias, 26 (41.9%) had organic acidemias and nine (14.5%) had FAO disorders. Parental consanguinity was found in one patient with 3-hydroxy-3-methylglutaryl (HMG) CoA lyase deficiency, and a family history of IEM was reported in three patients.

### Aminoacidemias

The most common aminoacidemia was PKU (11 patients, 40.7%), followed by maple syrup urine disease (MSUD) (5 patients, 18.5%), NICCD (5 patients, 18.5%), homocystinuria, and OTC deficiency (Table 2). Seven of the 11 PKU patients presented with language and motor development delays, and three had convulsions and epilepsy when referred for diagnosis. One of the 11 PKU patients showed normal growth and development, but the others still had growth retardation after treatment. All patients with MSUD showed poor appetite, convulsions, septicemia, irritability, and lethargy shortly after birth. Parents of two patients with MSUD refused treatment for their children after diagnosis. All five patients with NICCD had infant hepatitis syndrome at diagnosis, presenting with jaundice and abnormal hepatic function tests, though all developed well with normal hepatic function tests after treatment. Two patients with homocystinuria presented with jaundice at diagnosis; one of them developed normally with normal laboratory results after treatment, but the other was lost to follow-up. Two of the three patients with OTC deficiencies discontinued treatment after diagnosis, and the other one developed well after 6-months treatment with a protein-restricted diet, and arginine and citrulline supplementation.

**Table 2:** Abnormal MS/MS results of aminoacidemias

Aminoacidemias (n = 27)	n (%)	Age at diagnosis	Abnormal parameter	Concentration mean (range) (μmol/l)	Reference range (μmol/l)
Phenylketonuria	11 (40.7%)	1.4-135.6 mon	Phe	798.80 (216-1229)	28.08-103.18
			Phe/Tyr	9.01 (2.02-19.87)	0.15-3.0
Maple syrup urine disease	5 (18.5%)	2-26 d	Leu	3,390.57 (2,832.99-4,098.79)	88.26-327.51
			Val	600.51 (358-883)	89.5-433.56
Neonatal intrahepatic cholestasis caused by citrin deficiency	5 (18.5%)	2-4 mon	Cit	219.7 (89-318)	6.05-37.35
Homocystinuria	3 (11.11%)	0.6-36 mon	Met	335.5 (100-626)	10.82-64.11
Ornithine transcarbamylase deficiency	3 (11.11%)	0.07-7 mon	Cit	5.28 (5.15-5.45)	6.05-37.35
			Orn	398.33 (312-452)	47.53-393.08

### FAO disorders

Nine patients had FAO disorders, of which PCD was the most common (8/9, 89%) (Table 3). Convulsions were the most obvious symptom in these patients, and one presented with cardiomyopathy. Cardiac and neurological symptoms disappeared rapidly in these patients after supplementation with L-carnitine, with no occurrence of metabolic disorders or sudden death. The one patient detected with medium-chain acyl-CoA dehydrogenase (MCAD) deficiency was a girl, aged 26 months at diagnosis. Her initial presentation was febrile convulsions, and her blood C6, C8, C10 levels were elevated at screening. She developed normally with normal biochemical analysis after treatment with oral carnitine and standard diet recommendation.

**Table 3:** Abnormal MS/MS results of fatty acid oxidation disorders

Fatty acid oxidation disorders (n = 9)	n (%)	Age at diagnosis	Abnormal parameter	Concentration mean (range) (μmol/l)	Reference range (μmol/l)
Primary carnitine deficiency	8 (89%)	0.6-89 mon	C0	9.7 (0.87-14.10)	15.0-95.03
			C2	5.5 (2.3-6.6)	9.82-39.9
			C6	0.49	0.0-0.37
Medium-chain acyl-CoA dehydrogenase	1 (11%)	2 mon	C8	0.54	0.0-0.33
			C10:1	0.55	0.0-0.33

### Organic acidemias

Methylmalonic acidemia (MMA) was the most common organic acidemia in this cohort of patients, followed by propionic acidemia (PA), MCD and glutaric acidemia type 1 (GA-I) (Table 4). Other types of organic acidemia were rare in these patients. All patients with MMA and PA presented with hypoglycemia, metabolic acidosis, convulsions and developmental delay. Two MMA patients died, and the parents of the other two discontinued treatment. Two of the four PA patients died, one from metabolic acidosis and the other from respiratory failure. The symptoms in the patients with GA-I varied; two presented with recurrent convulsions and motor developmental delay at 1 year of age, while the other had macrocephaly and hypotonia at 4 months of age. Cranial magnetic resonance imaging showed extensive abnormal signals in the white matter and basal ganglia, ventriculomegaly and frontotemporal atrophy, widened sylvian fissures (bat-wing appearance). All three patients improved remarkably after 11, 13 and 16 months of follow-up, respectively. The patient with HMG-CoA lyase deficiency died soon after diagnosis.

**Table 4:** Abnormal MS/MS results of organic acidemias

Organic acidemias (26)	n (%)	Age at diagnosis	Abnormal parameters	Concentration mean (range) (μmol/l)	Reference range (μmol/l)
Methylmalonic academia	12 (46.2%)	0.1-99 mon	C3	10.3 (5.1-28.77)	0.47-4.33
			C3/C2	0.79 (0.11-1.88)	0.03-0.10
Propionic acidemia	4 (15.4%)	0.27-28 mon	C3	12.41 (8.27-13.8)	0.47-4.33
			C3/C2	1.31 (0.57-2.47)	0.03-0.10
			C5OH	3.88 (2.12-5.64)	
Multiple carboxylase deficiency	4 (15.4%)	5-60 mon	C3/C2	0.3 (0.290.31)	0.0- ^-^0.73
			C3	6.58 (4.32-6.84)	
Glutaric acidemia type I	3 (11.5%)	17-48 mon	C5DC	1.84 (0.78-3.58)	0.03-0.14
Isovaleric acidemia	2 (7.7%)	0.27-48 mon	C5	6.78 (4.21-9.35)	0.0-0.69
HMG-CoA lyase deficiency	1 (3.8%)	8 mon	C5OH	10.48	0.0-0.73

## Discussion

The introduction of MS/MS into neonatal screening has enabled the screening of conditions that might otherwise have been missed, and thus believed to be extremely rare [3,4]. This technique has significantly improved the efficacy of neonatal screening programs, demonstrating the importance of early identification and treatment of infants with disorders that would otherwise go unrecognized, before irreversible clinical damage occurs [5,6].

A total of 62 of 11,060 symptomatic patients (0.56%) were diagnosed with IEM in our study, which was higher than the percentage in a Korean study [7], which diagnosed 20 out of 6,795 symptomatic children with IEM (0.29%). However, several other studies [8,9] including one Indian and two Chinese studies, reported even higher detection rates of 3.2%, 6.6% and 9.6%, respectively. The wide variation in detection rates is not surprising, given the different screening criteria for IEM used in different countries, and the inconsistent sample-collection methods. Samples in the study by Gu et al. [9]. Included patients highly suspected of metabolic diseases from throughout the country, while most of our samples were from outpatients and inpatients in a single hospital. The detection rate in our series was thus much lower.

Amino acid disorders in our study accounted for 43.5% of total cases, with PKU being the most common type. This proportion was similar to two previous studies [6,7]. All the HPA patients were of classical type, and no case of BH_4 _deficiency was found. The age at diagnosis of the PKU patients was much older than in other reports [8], with a median age of 32 months (range: 1.3-135 months). Most patients had mental and language developmental delays at diagnosis. Apart from one patient diagnosed at 1 month of age who showed normal mental development after treatment, the remaining 10 patients suffered from mental retardation during follow-up, possibly as a result of older age at diagnosis. All the PKU patients were from the neighboring provinces including Anhui and Jiangxi Provinces. Although newborn screening for PKU and congenital hypothyroidism have been implemented for 30 years with a coverage rate of 97.5% in Zhejiang Province, the average coverage rate for newborn screening was still below 50% over the whole country in 2010. PKU patients may therefore not be detected at an early age in the regions with lower coverage rates, which could explain why all the PKU patients were from Anhui and Jiangxi Provinces, which have low newborn screening coverage rates. Mental retardation is difficult to reverse in children detected by symptomatic screening at an older age, indicating the need to improve the newborn screening coverage rate throughout China.

The second most common disease in this study was MSUD, as in other reports in Asian populations [4,8]. Although diagnosed at an early age (< 1 month), MSUD patients commonly become symptomatic 4-5 days after birth and neuropathological symptoms occur very shortly after. Death may occur in patients without standard treatment. The patients in our study presented with neurological symptoms such as poor feeding, dystonia, poor response and somnolence, and cranial magnetic resonance imaging revealed abnormal signals in the thalamus, brainstem and cerebellum at diagnosis. Three patients showed mental and motor development delays after treatment with a low-protein diet and special amino acid formula. Treatment was discontinued by the parents in two patients because of financial problems or worry about the poor outcome. MSUD had the poorest outcome compared with other aminoacidemias, while NICCD had the best; all NICCD patients recovered, with normal liver function, after treatment with lactose-free milk powder and other medications.

Similar to other reports [6,10], FAO disorders were uncommon in the Asian population. PCD was the most common type of FAO disorder in the current study. Most PCD patients were identified between 1 month and 7 years of age, and all but one of the PCD patients initially presented with convulsions. PCD may have a good outcome if detected at early age and given timely treatment. MCAD has been reported to be the most common type of FAO in Europe and USA, and its incidence was even higher than that of the aminoacidemias. However, only one case of MCAD was found during the present 3-year screening study, similar to the report by Han et al., from Shanghai [11]. Other types of FAO disorders are also rarely found in the Chinese population. However, blood sampling was not performed under strictly fasting conditions for most children, and patients with some types of FAO may have been missed, thus underestimating the incidence of FAO.

Organic acidemias accounted for 41.9% of IEM cases in this study, with MMA, PA and MCD being the three most common types. PA and MMA should be differentiated by GC-MS because of their similar biochemical results, while elevated C3 and C3/C2 ratios are much higher in PA patients than in MMA patients. MCD had the best outcome, and all four MCD patients recovered dramatically after treatment with oral biotin, with no mental developmental delay. Mental retardation persisted, however, in the patients with PA and MMA despite treatment. All GA-I patients had motor developmental delay. The baby with HMG CoA lyase deficiency deteriorated rapidly and died before diagnosis. Our study indicated that irreversible neurological sequelae were likely to occur if patients with organic acidemias were not diagnosed and treated at an early stage of the disease. Newborn screening for IEM is thus imperative.

The major limitation of this study was the cut-off values used for screening symptomatic children. Because these cut-off values were based on newborns, some cases in the study population, with an age range from 0.04-168.2 months, may have been missed. Age-specific cut-off values need to be established in further studies. According to the report by McHugh et al. [12]. Further validation of the cut-offs will ensure a more accurate and early diagnosis of IEM.

The small number of diagnosed IEM patients in China means that the experience of their treatment is still very limited, and no uniform treatment guidelines have been established. Poor medical compliance occurs in most patients; treatment was discontinued by their parents in nine children as a result of economic problems and loss to follow-up. Of the remaining 53 patients, most still had various symptoms, including convulsions, motor and mental developmental delays, and language delays. Only 10 patients become asymptomatic with normal physical and mental development during the follow-up period, and these 10 had all been diagnosed at a much earlier stage and received timely treatment. This provides evidence for the importance of expanded newborn screening throughout the province. Local governments should consider including expanded newborn screening in free healthcare coverage.

## Conclusions

The patients in this study were all symptomatic at screening, and most of them were beyond the neonatal period. Because mental retardation and motor delay are difficult to reverse in older patients with IEM, it is essential to increase the availability of newborn screening. Additionally, IEM patients require life-long treatment, and the associated costs might thus represent a heavy burden for families with low or even middle social economic incomes. Lack of compliance with recommended treatments and medication dosages may lead to poorer outcomes. The government should consider increasing insurance coverage for patients with IEM in order to improve patient compliance and consequent treatment efficacy.

## Competing interests

The authors declare that they have no competing interests.

## Authors' contributions

XH and LY conceived and designed the study and acquired the data. All authors were involved in the analysis and interpretation of data and drafting and revision of the manuscript. All authors read and approved the final manuscript.

## Authors' information

Dr Xinwen Huang is the vice director of the Department of Genetics and Metabolism, Children's Hospital, who is an associate professor of Pediatrics experienced in diagnosis and treatment of children with inborn errors of metabolism.

## Funding

This article was partly supported by the National Natural Science Foundation of China (491040-N11157), Family Planning Commission of Zhejiang Province (491040-WO1103), Zhejiang Province innovation team for early screening and intervention of birth defects (2010R50045) and Hall of Zhejiang Province Science and Technology (2011C33G2010350).

## Pre-publication history

The pre-publication history for this paper can be accessed here:

http://www.biomedcentral.com/1471-2431/12/18/prepub
